# The H3K9 Methylation Writer SETDB1 and Its Reader MPP8 Cooperate to Silence Satellite DNA Repeats in Mouse Embryonic Stem Cells

**DOI:** 10.3390/genes10100750

**Published:** 2019-09-25

**Authors:** Paola Cruz-Tapias, Philippe Robin, Julien Pontis, Laurence Del Maestro, Slimane Ait-Si-Ali

**Affiliations:** 1 Epigenetics and Cell Fate (EDC), Centre National de la Recherche Scientifique (CNRS), Université de Paris, Université Paris Diderot, F-75013 Paris, Francephilippe.robin36@gmail.com (P.R.); julien.pontis@epfl.ch (J.P.); laurence.del-maestro@univ-paris-diderot.fr (L.D.M.); 2 Faculty of Natural Sciences and Mathematics, Universidad del Rosario, Bogotá 111221, Colombia; 3 School of Medicine and Health Sciences, Universidad del Rosario, Bogotá 111221, Colombia; 4 Doctoral Program in Biomedical and Biological Sciences, Universidad del Rosario, Bogotá 111221, Colombia

**Keywords:** M-Phase Phosphoprotein 8, SETDB1/KMT1E, histone lysine methylation, satellite DNA repeats, transcription

## Abstract

SETDB1 (SET Domain Bifurcated histone lysine methyltransferase 1) is a key lysine methyltransferase (KMT) required in embryonic stem cells (ESCs), where it silences transposable elements and DNA repeats via histone H3 lysine 9 tri-methylation (H3K9me3), independently of DNA methylation. The H3K9 methylation reader M-Phase Phosphoprotein 8 (MPP8) is highly expressed in ESCs and germline cells. Although evidence of a cooperation between H3K9 KMTs and MPP8 in committed cells has emerged, the interplay between H3K9 methylation writers and MPP8 in ESCs remains elusive. Here, we show that MPP8 interacts physically and functionally with SETDB1 in ESCs. Indeed, combining biochemical, transcriptomic and genomic analyses, we found that MPP8 and SETDB1 co-regulate a significant number of common genomic targets, especially the DNA satellite repeats. Together, our data point to a model in which the silencing of a class of repeated sequences in ESCs involves the cooperation between the H3K9 methylation writer SETDB1 and its reader MPP8.

## 1. Introduction

Post-translational modifications of histones play key roles in DNA functions in the chromatin context. In this, methylation of histone H3 lysine 9 (H3K9) generally correlates with transcriptional repression and heterochromatin formation [1]. The main location of H3K9 methylation is on heterochromatin and more generally on repetitive elements, such as the major and minor satellite repeats in the mouse genome [1]. Highly condensed heterochromatin regions are enriched in trimethylated H3K9 (H3K9me3), whereas euchromatin regions are preferentially enriched in mono- and di-methylated H3K9 (H3K9me1 and H3K9me2, respectively) [2].

SET Domain Bifurcated histone lysine methyltransferase 1 (SETDB1 or ESET in mouse) is a key H3K9 lysine methyltransferase (KMT). SETDB1 is able to establish H3K9 mono, di- and tri-methylation, the latest in cooperation with its co-factor ATF7IP (Activating Transcription Factor 7-Interacting Protein 1, also called MCAF1), which is necessary for the conversion of H3K9me2 to H3K9me3 [3]. Thus, this ability of SETDB1 to establish the three H3K9 methylation levels makes this KMT important in both euchromatin and heterochromatin. SETDB1 is essential in mouse embryonic stem cells (mESCs) pluripotency and self-renewal [4,5,6] and its knockout (KO) is lethal at the peri-implantation stage at 3.5 dpc [7]. *Setdb1*-null mouse blastocysts fail to give rise to ESCs in vitro [7] and *Setdb1* knockdown (KD) in mESCs results in loss of *Oct4* expression and abnormal expression of various differentiation markers and de-repression of many repeated elements [6,8]. Consistently, SETDB1 has been found to occupy and silence trophoblastic and developmental genes, and retroviruses in mESCs [4,5,8,9,10].

Methylated lysine residues serve as docking sites for numerous proteins that contain methyl-lysine-binding domains such as the chromodomain, the Plant HomeoDomain (PHD) or the Tudor domains [11]. Among these readers of methylated lysines, M-Phase Phosphoprotein 8 (MPP8) has been shown to bind methylated H3K9 [12,13,14]. MPP8, which was first characterized as a M-phase phosphoprotein [15], harbors two main functional domains, a well-characterized N-terminal chromodomain (chromobox) and an ankyrin repeat domain, with unknown function, in the C-terminal part. Notably, the chromobox binds methylated lysines, especially di- or trimethylated H3K9 [12,13,14].

Expression of MPP8 is especially high in stem and germ cells and thus may play key roles in these cells’ chromatin features. However, its role in ESCs has never been studied, contrary to the main H3K9 KMTs SETDB1, G9A/GLP and SUV39H1/2 that are extensively studied in mESCs (reviewed by Mozzetta, et al. in [1]).

Here, we have combined biochemical and genomic strategies to get insights on the functions of the major H3K9 reader MPP8 in mESCs, where H3K9 methylation plays key roles in the non-coding genome and transposable elements silencing compared to committed cells. Our TAP-tag assay showed that MPP8 co-purifies with many H3K9 KMTs, with a higher score for SETDB1. Interaction of endogenous MPP8 and SETDB1 was confirmed in mESCs. Combined ChIP-seq and RNA-seq unraveled that MPP8 cooperates with SETDB1 to co-regulate repeated elements, including the minor and major satellite DNA repeats. Our results suggest a new regulatory mechanism for repeated sequences in mESCs which involves the cooperation between the major H3K9 methylation writer SETDB1 and its reader MPP8.

## 2. Materials and Methods

### 2.1. Cell Culture

HeLa-S3 cell line was grown in Dulbecco’s modified Eagle’s Medium DMEM (Sigma; St. Louis, Missouri; USA) supplemented with 10% of fetal bovine serum (FBS-GE), 1% penicillin/streptomycin (Sigma; St. Louis, Missouri; USA) and sodium pyruvate (Sigma; St. Louis, Missouri; USA). HeLa-S3 cells were maintained at 37 °C and 5% CO_2_.

HM1 and TT2 mESCs were cultured in ESCs media: DMEM media (Sigma; St. Louis, Missouri; USA) supplemented with 15% fetal calf serum (Gibco; Waltham, Massachusetts, USA), 1% penicillin/streptomycin (Sigma; St. Louis, Missouri; USA), 0.1 mM β-mercaptoethanol (Thermo-Fisher Scientific; Waltham, Massachusetts, USA), 1 mM nonessential amino acids (Sigma; St. Louis, Missouri; USA), and 1000 U/mL of Leukemia Inhibitory Factor (LIF) (Millipore; Burlington, Massachusetts; USA). All mESC lines were cultured in standard feeder-free conditions with 0.2% gelatin, maintained at 37 °C and 8% CO_2_.

*Setdb1* cKO mESCs were established by the group of Prof. Yoishi Shinkai via standard gene targeting procedures [16]. To generate the *Setdb1* cKO mESC line, Cre recombinase and estrogen receptor (Cre-ER) fusion gene was introduced into a clone containing targeted *Setdb1* cKO and KO alleles. To induce deletion of the *Setdb1* cKO allele, TT2 mESCs were cultured in 800 nM 4-Hydroxy Tamoxifen (Sigma; St. Louis, Missouri; USA) for 96 hours.

### 2.2. siRNA Transfection

HM1 ESCs were seeded in ESCs media to achieve 70–80% confluence and 24 h later were transfected with siRNAs (SETDB1, MPP8 and non-targeting scrambled control) to achieve a final siRNA concentration of 25 pM using the Lipofectamine RNAiMax reagent and OPTIMEM (Life technologies; Waltham, Massachusetts, USA). A second round of transfection was performed 24 h later using the same siRNA concentration. Cells were harvested after 72 h post first transfection. siRNA sequences are listed in the Table 1.

### 2.3. Protein Complex Immuno-Purification

We used HeLa-S3 cell lines stably expressing Flag-HA tagged MPP8 (only chromobox or full-length), established by retroviral transduction of human full-length transgenes, as described in [17]. A cell line transduced with the empty pREV vector has been used as control. We carried out double-affinity purification of Flag-HA-MPP8 from HeLa cells using Flag (Cat# F7425; Sigma; St. Louis, Missouri; USA) and HA (Cat# 3F10; Roche; Basel, Switzerland) antibodies and either nuclear soluble or chromatin fractions as described in [17]. Double-immunopurified complexes were resolved on 4–12% SDS-PAGE bis-Tris acrylamide gradient gel in MOPS buffer (Invitrogen; Waltham, Massachusetts, USA) and analyzed by mass spectrometry and Western blot.

### 2.4. Immunoprecipitation (IP)

40 million cells were lysed in Buffer A (20 mM HEPES pH 7, 0.15 mM EDTA, 0.15 mM EGTA, 10 mM KCl), 10% NP40 and SR buffer (50 mM HEPES pH 7, 0.25 mM EDTA, 10 mM KCl, 70% (m/v) sucrose) supplemented with protease inhibitor (Sigma; St. Louis, Missouri; USA), 1 mM DTT and spermidine-spermine (0.15 mM, 0.5 mM, respectively) to limit nuclei leak. Cell lysates were centrifuged at 2000 g for 5 min. The nuclei pellets were resuspended in sucrose buffer (20 mM Tris pH 7.65; 60 mM NaCl; 15 mM KCl; 0.34 M Sucrose) and high salt buffer was added (20 mM Tris-HCl pH 7.65; 0.2 mM EDTA; 25% glycerol; 900 mM NaCl; 1.5 mM MgCl_2_) to a final NaCl concentration of 300 mM, and incubated for 30 min on ice. Then, sucrose buffer was added to a final NaCl concentration of 100 mM. The nuclear extracts were treated with Micrococcal nuclease (0.0125 U/mL) and 1 mM CaCl_2_ at 37 °C during 15 min. Then, EDTA was added to 4 mM final concentration and subsequent sonication for 5 min (15 sec ON, 1 min OFF) at medium frequency (Bioruptor Diagenode; Liège, Belgium) was performed. The lysates were ultracentrifugated at 40000 rpm for 30 min and pre-cleared with protein G-agarose beads (Sigma; St. Louis, Missouri; USA) during 2 h, at 4 °C. Immunoprecipitations were carried out overnight at 4 °C using 500 μg of nuclear extract and 5 μg of antibodies against SETDB1 (Cat# sc-66884; Santa Cruz Biotechnologies; Dallas, Texas; USA), MPP8 (Cat# 16796-1-AP; Proteintech; Rosemont, Illinois; USA) and IgG (Cat# A0545; Sigma; St. Louis, Missouri; USA). Pierce™ Protein A/G UltraLink™ Resin (Thermo-Fisher Scientific; Waltham, Massachusetts, USA) was blocked overnight at 4 °C with 0.3% Bovine Serum Albumin (BSA). Immune complexes were recovered by adding pre-blocked protein A/G Ultralink beads and incubated for 2 h, at 4 °C. Beads were washed four times in Wash buffer (50 mM Tris-HCl pH 8.0; 150 mM NaCl; 0.5% Triton X-100) and the proteins were eluted in NuPAGE® LDS Sample Buffer (Life Technologies; Waltham, Massachusetts, USA) and 10X reducing agent at 96 °C during 10 min. Finally, the immunoprecipitates were tested by Western blot.

### 2.5. Western Blot

Nuclear extracts or immunoprecipitates were resolved on pre-cast polyacrylamide gel cassettes (NuPAGE® Novex® 4–12% Bis-Tris) (Invitrogen; Waltham, Massachusetts, USA) and 1X NuPAGE MES SDS Running Buffer and transferred into nitrocellulose membrane (Amersham; Chicago, Illinois; USA) in 20 mM phosphate transfer buffer (pH 6.7). Membrane was blocked in 5% skim milk in PBST Buffer (1X PBS, 0.2% Tween 20) and incubated overnight at 4 °C with the primary antibodies against SETDB1 (Cat# sc-66884; Santa Cruz Biotechnologies; Dallas, Texas; USA), MPP8 (Cat# 16796-1-AP; Proteintech; Rosemont, Illinois; USA). Membranes were incubated with the appropriate secondary antibody coupled to HRP, revealed using West Dura kit (Pierce, Rockford, USA) and ChemiSmart 5000 system (Vilber Lourmat; Marne-la-Vallée; France). When necessary, the TrueBlot secondary Ab (Clinisciences; Nanterre; France) was used to reduce interference with the ~55 kDa heavy and ~23 kDa light chains of immunoprecipitating IgG.

### 2.6. Chromatin Immunoprecipitation (ChIP)

Cells were cross-linked directly in the culture plate with PBS supplemented with 1 mM MgCl_2_ and 2 mM DiSuccinimidyl glutarate (Thermo-Fisher Scientific; Waltham, Massachusetts, USA) diluted in DMSO (Sigma; St. Louis, Missouri; USA) during 45 min at RT as described in [18]. Then, a second crosslinking was carried out by adding 1% formaldehyde (culture medium supplemented with 1% formaldehyde (Sigma; St. Louis, Missouri; USA), 15 mM NaCl, 0.15 mM EDTA, 0.075 mM EGTA, 0.015 mM Hepes pH 8.0) during 10 min at RT. Formaldehyde was quenched with 0.125 M glycine and cells were washed in PBS and pelleted. Fixed cells were then incubated on the wheel at 4 °C for 10 min in Buffer 1 (50 mM Hepes/KOH pH 7.5; 140 mM NaCl; 1 mM EDTA; 10% Glycerol; 0.5% NP-40; 0.25% Triton X-100). After centrifugation, cells were incubated on the wheel at 4 °C for 10 min in Buffer 2 (200 mM NaCl; 1 mM EDTA; 0.5 mM EGTA; 10 mM Tris pH 8.0). Nuclei were then pelleted by centrifugation, resuspended in Buffer 3 (50 mM Tris pH 8.0; 0.1% SDS; 1% NP-40; 0.1% Na-Deoxycholate; 10 mM EDTA; 150 mM NaCl), and sonicated for 20 min (30 sec ON, 30 sec OFF) (Bioruptor Diagenode; Liège, Belgium), yielding genomic DNA fragments with a bulk size of 150-600 bp. All buffers were supplemented with protease inhibitors prior to usage. Chromatin corresponding to 10μg of DNA was pre-cleared with protein G-agarose beads (Sigma; St. Louis, Missouri; USA) during 2 h, at 4 °C. 1% of chromatin extracts were taken aside for inputs. Immunoprecipitations with anti-MPP8 antibody (Cat# 16796-1-AP; Proteintech; Rosemont, Illinois; USA) were carried out overnight at 4 °C. Pierce™ Protein A/G UltraLink™ Resin (Thermo-Fisher Scientific; Waltham, Massachusetts, USA) was blocked overnight at 4 °C with 0.3% BSA. Immune complexes were recovered by adding pre-blocked protein A/G ultralink beads and incubated for 2 h, at 4 °C. Beads were washed twice with Low salt buffer (0.1% SDS; 1% Triton; 2 mM EDTA; 20 mM Tris pH 8.0; 150 mM NaCl), twice with High salt buffer (0.1% SDS; 1% Triton; 2 mM EDTA; 20 mM Tris pH 8.0; 500 mM NaCl), once with LiCl wash buffer (10 mM Tris pH 8.0; 1% Na-deoxycholate; 1% NP-40, 250 mM LiCl; 1 mM EDTA), and once with TE supplemented with 50 mM NaCl. Cross-linked chromatin was eluted in TE with addition of 1% SDS and 0.2 M NaCl at 65 °C during 45 min. ChIP-enriched samples and inputs were then reverse cross-linked at 65 °C overnight and treated with 0.3 μg/mL RNase A. Eluted material was incubated with Proteinase K 2 h at 37 °C. The eluted material was phenol/chloroform-extracted and ethanol-precipitated.

### 2.7. ChIP-Sequencing (ChIP-Seq)

Five to fifteen nanograms of ChIPed DNA or un-enriched whole cell extract (Input) were prepared for sequencing on Illumina Hiseq 2000. We used the library kit (Truseq DNA sample prep kit V2, Illumina; San Diego, California; USA) with modifications as follows: DNA fragments were repaired to blunt ends, purified with magnetic beads (Agencourt AMPure XP, Beckman coulter) and a step of A tailing was performed before Illumina adapters ligation. Two steps of DNA purification with magnetic beads were carried out to eliminate un-ligated adaptors and then amplified with 15 PCR cycles. To remove un-ligated adapters and un-sequenceable large DNA fragments, DNA libraries were selected on E-Gel (2% SizeSelect, Invitrogen; Waltham, Massachusetts, USA) to obtain 280–330 bp DNA fragments (including 130 bp of adapters). Final library was quality checked by DNA high sensitivity chip (Agilent; Santa Clara, California; USA) and for positive target enrichment by qPCR. Quality-controlled samples were then quantified by qPCR and picogreen (Qubit® 2.0 Fluorometer, Invitrogen; Waltham, Massachusetts, USA). Libraries were pooled thanks to various adaptors and qPCR relative measurements. Cluster amplification and following sequencing steps strictly followed Illumina standard protocol.

Sequenced reads were de-multiplexed to attribute each read to a DNA sample and then aligned to reference mouse genome mm10 with bowtie (-t -q -p 8 -S -n 2 -e 70 -l 50 --maxbts 125 -k 1 -m 1 --phred33-quals). After PCR duplicates removal, enriched regions were detected by MACS 1.4 software package [19], using Input DNA as a control. MACS 1.4 was used to visualize read enrichments. The BEDTools [20] “intersect” and “mergeBED” were used to filter and merge overlapping peaks between replicates. Merged peaks were assigned to the closest gene within a 10 Kb distance using the Ensembl annotation. Merged peaks were also assigned to overlapping repeats using the RepeatMasker annotation. Peaks visualization was carried out with the Integrative Genomics Viewer - Broad Institute [21].

### 2.8. RNA and Quantitative Reverse Transcription-PCR (RT-qPCR)

Total RNA was extracted using RNeasy mini-kit (Qiagen; Venlo, Netherlands) following manufacturer’s procedures. DNase (Qiagen; Venlo, Netherlands) treatment was performed to remove residual DNA. With High Capacity cDNA Reverse Transcription Kit (Applied Biosystems; Waltham, Massachusetts, USA), 1 μg of total RNA was reverse transcribed. Real-time quantitative PCR was performed to analyze relative gene expression levels using SYBR Green Master mix (Applied Biosystems; Waltham, Massachusetts, USA) following manufacturer indications. Relative expression values were normalized to the housekeeping genes mRNA *Cyclophillin* A or *GAPDH*. Primers are listed in the Table 1.

### 2.9. RNA-Sequencing (RNA-Seq)

RNA was isolated as described above. Three independent biological replicates were sequenced per cell condition. Libraries were generated using the TruSeq stranded mRNA library prep Kit, set A and B (Cat# RS-122-2101, Cat# RS-122-2102; Illumina; San Diego, California; USA). The obtained directional libraries were controlled by Bioanalyzer DNA1000 Chips (Cat# 5067-1504; Agilent; Santa Clara, California; USA) and quantified by spectrofluorimetry (QuantiT™ High-Sensitivity DNA Assay Kit, Cat#Q33120, Invitrogen; Waltham, Massachusetts, USA). Sequencing was performed on a HiSeq 2500 (Illumina; San Diego, California; USA) in a 51 bases single read using a HiSeq SR Cluster kit v4 cBot HS (Cat# GD-401-4001 Illumina; San Diego, California; USA) and a HiSeq SBS kit v4 HS 50 cycles (Cat# FC-401-4002; Illumina; San Diego, California; USA). Sequences were demultiplexed using the Illumina pipeline (Gerald, included in CASAVA version 1.8) giving FASTQ formatted reads. 

Using the Galaxy platform, high quality one-end reads for the three replicates (siRNA SETDB1, MPP8 and control) were mapped onto mouse genome (mm10) using Bowtie2 v2.3.4.1 [22] with default alignment parameters (-I 0 -X 500). *Mus musculus* GRCm38.9 GTF file was downloaded from Ensembl (https://www.ensembl.org) and the exon annotation lines were extracted from the file to use as GTF annotation file. FeatureCounts v1.6.0.2 [23] was used with this GTF file to treat the BAM alignment files and calculate the gene expression values in transcripts per million (TPM). Differentially expressed genes were identified using he DESeq2 Galaxy wrapper v2.11.40.6 [24]. mm10 repeat elements were downloaded from the RepeatMasker site (www.repeatmasker.org) and repeats annotated as “Low complexity” or “Simple repeat” were filtered out. RepEnrich [25] was used to annotate and count the reads aligned to repeats. The ARTbio Galaxy wrapper (edgeR-repenrich v1.5.3) of EdgeR [26] was used to identify differentially expressed repeats and repeat classes. The Database for Annotation, Visualization, and Integrated Discovery (DAVID) version 6.8 (https://david.ncifcrf.gov/) [27,28] was employed for Gene Ontology (GO) analysis.

### 2.10. Additional Statistical Analyses

The collected data was analyzed using Statistical Package for Social Sciences (SPSS Version 25, Chicago IL). Normality of the data was assessed using the Shapiro-Wilk test. Results were presented as mean and standard deviation (SD) or median and interquartile range (IQR). Differences between groups were compared using t-test or Mann Whitney’s unpaired test based on normality test. Statistical significance was assessed at the α < 0.05 levels for RT-qPCR.

### 2.11. Data Availability

All NGS sequencing data that support the findings of this study are available in the BioProject database of NCBI (https://www.ncbi.nlm.nih.gov/bioproject) under the accession number PRJNA565573.

### 2.12. Primer and siRNA Sequences

**Table 1:** Primer and siRNA sequences.

Targets	Forward	Reverse
**RT-qPCR**		
Cyclophillin A	GTCAACCCCACCGTGTTCTT	CTGCTGTCTTTGGGACCTTGT
GAPDH	CCAATGTGTCCGTCGTGGATCT	GTTGAAGTCGCAGGAGACAACC
SETDB1	GCCAAAGGCTCTTTTGTCTG	TCAGAGGAAGTGGGGACATC
MPP8	GTGAAGGTTGCACTGAACTC	TCTCAGCGGCATGAATGAGG
LINE	TTTGGGACACAATGAAAGCA	CTGCCGTCTACTCCTCTTGG
Major Sat	GACGACTTGAAAAATGACGAAATC	CATATTCCAGGTCCTTCAGTGTGC
Minor Sat	CATATTCCAGGTCCTTCAGTGTGC	GTTCTACAAATCCCGTTTCCAAC
**siRNA**		
SETDB1	GUGGAAGUCUCGAGUUGAA dTdT	UUCAACUCGAGACUUCCAC dTdT
MPP8	CAAGGUUAAGUUGCUAAUA dTdT	UAUUAGCAACUUAACCUUG dTdT
Scrambled	GCCGGUAUGCCGGUUAAGU dTdT	ACUUAACCGGCAUACCGGC dTdT

## 3. Results

### 3.1. MPP8 Protein Complex Characterization

**Figure 1:**
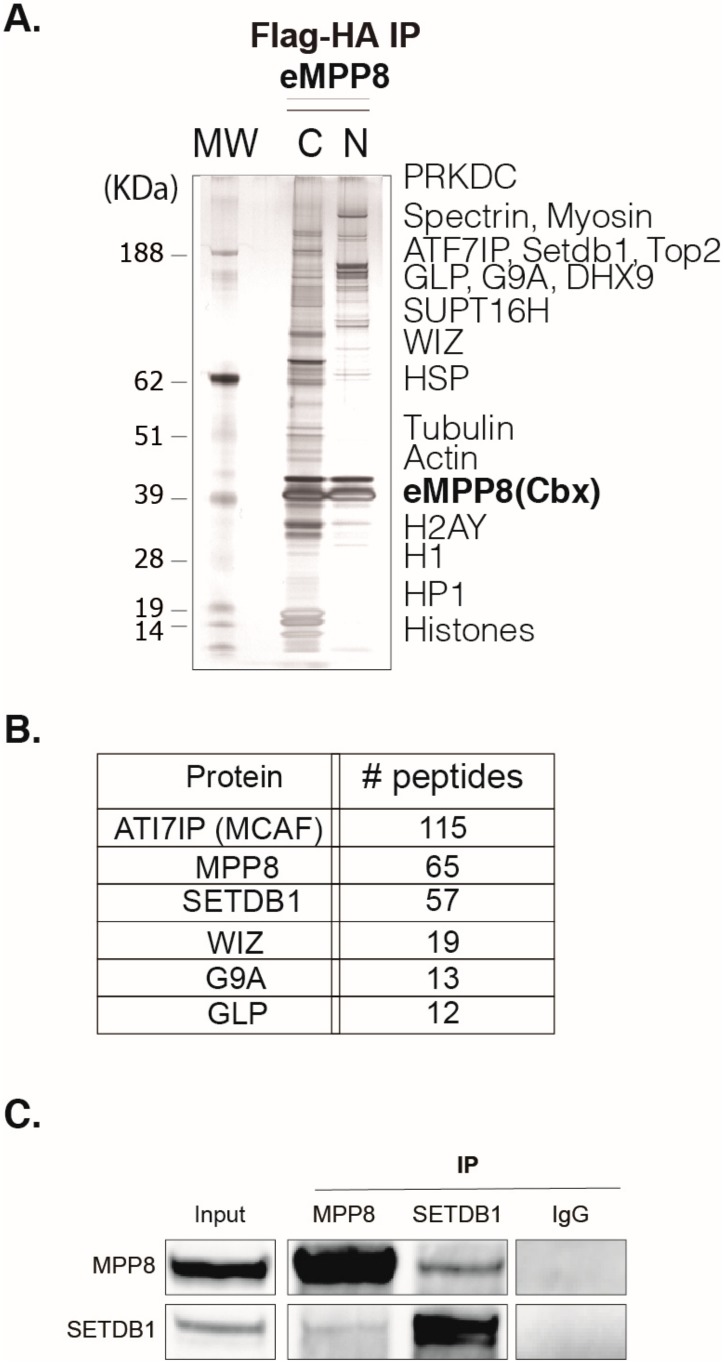
MPP8 complex characterization. (**A**) Silver staining of MPP8 complex. The double immuno-purification of MPP8-choromobox complex has been performed from either chromatin-enriched (C) or nuclear soluble (N) extracts of HeLa-MPP8-chromobox cells. Eluted eMPP8 (cbx) complexes were resolved on a 4–12% gradient of Sodium dodecyl sulfate polyacrylamide gel electrophoresis (SDS-PAGE). Note that we have loaded ten times more eMPP8 soluble complex (N) than chromatin-associated complex (C). MW, molecular weight marker, in KDa. (**B**) Scores of the top most abundant peptides as identified by MS. (**C**) Endogenous MPP8 and SETDB1 interact in mESCs. Nuclear extracts from HM1 mESCs were used for immunoprecipitation (IP) with the indicated Abs; IgG was used as negative control. The resulting precipitates were then subjected to western blot (WB) with indicated antibodies.

To investigate MPP8 functions, we first sought to globally identify its protein partners using a Tandem Affinity Purification (TAP)-tag strategy coupled to mass spectrometry (MS), as previously described (Fritsch et al., 2010). To this end, we carried out HA-Flag double-affinity immuno-purification from either nuclear soluble or nucleosome-enriched protein fractions (Figure 1A and Supplementary Figure S1A) of HeLa cells stably expressing MPP8 chromobox (MPP8cbx). MS and Western blot (WB) analyses showed that MPP8 is associated with proteins linked to H3K9 methylation, such as many H3K9 KMTs and effector proteins, but also scaffold proteins from the nuclear architecture, proteins involved in the RNA processing, chromatin remodeling and DNA repair (Table S1).

The H3K9 KMTs G9A, GLP and SETDB1 co-purified robustly with MPP8 (Figure 1B). Interestingly, the most abundant MPP8 partners were SETDB1 and its co-factor ATFIP7 (Figure 1B), which cooperates with SETDB1 to establish H3K9 tri-methylation.

In addition to the H3K9 methylation machinery, the MPP8 complex contained also DNA repair and maintenance machineries (XRCC5, XRCC6, TOP1, TOP2A, RCC1, DNA-PK and LIG3), chromatin remodeling complexes (SMARCA1, RSF), RNA maintenance and processing factors (hnRNPK, hnRNPH2, hnRNPM, hnRNPP2, DDX17, DDX21, DHX9, RBM39, KPNA2, SAM68, TARDBP, YBX1, RBM14 and LUC7L2), these proteins belong to the transcription process and RNA modification (Table S1).

To get more insights on MPP8 functions within chromatin, we next sought to identify post-translational modifications (PTMs) on the histones co-purified with MPP8 by in-gel propionylation and trypsin digestion and Nano LC coupled to Ion Trapp mass spectrometer, as described in [29]. Regarding H3K9 methylation, our results show that MPP8 co-purified mainly with H3K9me2 (56%) and H3K9me3 (44%) (Supplementary Figure S1B), in accordance with previous results [29]. SETDB1 complex purification from HeLa cells did not co-precipitate enough histones to perform such analysis [29]. While G9A co-purified mostly with H3K9me1 and H3K9me2, as expected [29]. Concerning H3K36 methylation, which is associated with transcription elongation, we found in association with MPP8 43% of unmethylated H3K36, 17% of H3K36me1, 39% of H3K36me2 and only 1% of H3K36me3. Compared to the KMT G9A, we found 48% of unmethylated H3K36, 30% of H3K36me1, 20% of H3K36me2 and 2% of H3K36me3 (Supplementary Figure S1B). Thus, there is less H3K36me1 and more H3K36me2 in association with MPP8 compared to G9A, suggesting that MPP8 is recruited to active genes.

Next, we decided to focus on the significance of the MPP8/SETDB1 interaction. To this end, we performed experiments in a more physiological cellular model, namely mouse embryonic stem cells (mESCs). Indeed, MPP8 is known to be highly abundant in ESCs, where SEDTB1 and H3K9 methylation play key roles in transcriptional silencing, in the absence of a complete establishment of DNA methylation before cell differentiation.

Thus, to further support our findings, we sought to confirm the interactions revealed by TAP-tag/MS at the endogenous level by performing co-immunoprecipitation (co-IP) experiments from nuclear extracts of mESCs, followed by WB analyses. Our results showed that immunoprecipitation of MPP8 co-precipitated SETDB1 (Figure 1C). Conversely, SETDB1 co-precipitated with MPP8 (Figure 1C). We next studied the functional significance of such interactions.

### 3.2. MPP8 and SETDB1 Co-Bind Repetitive Elements

To search for MPP8/SETDB1 common regulated genomic regions, we performed endogenous MPP8 ChIP-seq in mESCs (from the 129S1 mouse strain) and analyzed an already published SETDB1 ChIP-seq in mESCs (GSE18371; [4]).

**Figure 2:**
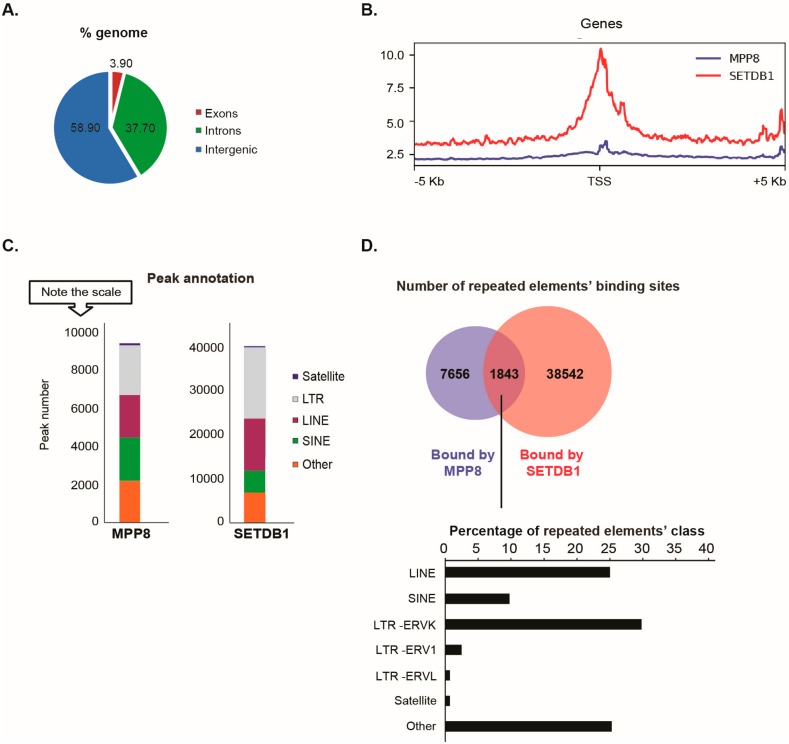
MPP8 and SETDB1 occupy common genes and repetitive elements genome-wide in mouse ESCs. (**A**) Venn Diagram of the genome-wide distribution of MPP8 binding sites in mESCs. (**B**) Global SETDB1 occupancy over MPP8 binding sites in mESCs. The graph represents average binding density of genomic regions surrounding (±5 kb) MPP8 and SETDB1 binding sites in mESCs. (**C**) Distribution of MPP8 and SETDB1 annotated peaks for the main families of retrotransposons in mESCs. Data represent the number of annotated peaks. (**D**) Venn diagram representation of transposable elements enriched in the binding sites of MPP8 and SETDB1 in mESCs. For co-bound sites, a representation of distribution of repeated elements’ classes is shown in percentages.

Our data showed that the distribution of MPP8 binding sites on the genome is almost 60% on intergenic regions (including repeated elements), 37% on introns and 4% on exons (Figure 2A).

Since MPP8 co-purified with the H3K9 KMT SETDB1, we next studied the extent of MPP8 co-localization with SETDB1. We found MPP8 co-localization on 6.5% binding sites with SETDB1 (Figure 2B). We found that 1916 genes are bound by MPP8. Interestingly, we observed co-localization of MPP8 and SETDB1 genome-wide on 1329 genes, which are mainly involved in nucleosome assembly (Supplementary Figure S2 and Supplementary Table S2). Concerning transposable elements analyses, MPP8 peaks were proportionally located in LTR and LINE and SINE repeats, which is similar to the binding sites reported for SETDB1 (Figure 2C). A total of 9499 transposable elements enriched in the binding sites of MPP8 were found, among which around 20% (1843/9499) are also bound by SETDB1 (Figure 2D and Supplementary Table S3). Interestingly, MPP8 and SETDB1 co-bind more LINE and ERVK elements but also (peri-)centromeric satellite DNA repeats including GSAT_MM, SYNREP (Figure 2D).

Altogether, the ChIP-seq data suggest that MPP8 cooperate with SETDB1 in transposable elements and satellite DNA repeats silencing in mESCs.

### 3.3. Regulation of Gene Expression by MPP8 and SETDB1 in mESCs

In light of the co-immunoprecipitation and ChIP-seq results described above, showing a physical interaction between MPP8 and SETDB1 and genomic co-binding in mESCs, we further investigated the possible functional interplay between MPP8 and SETDB1. We asked whether MPP8 and SETDB1 co-regulate gene expression in mESCs. To further confirm the functional overlap between MPP8 and SETDB1, we performed RNA-seq analyses in HM1 ESCs after *MPP8* KD or *SETDB1* knockdown (KD). RNA-seq data were further analyzed by differential expression pattern of genes and transposable elements using the scrambled siRNA condition as a control on the whole dataset.

**Figure 3:**
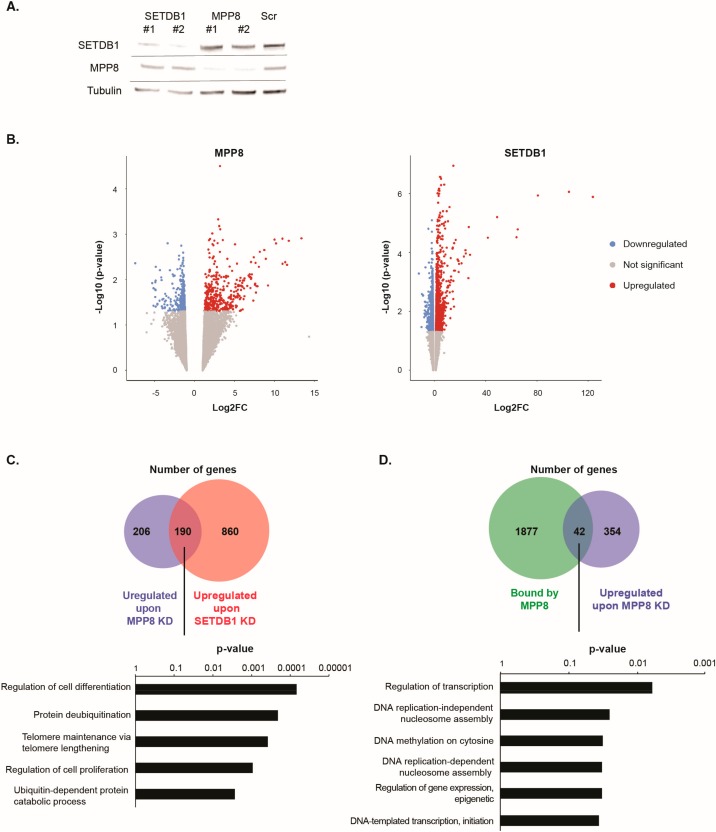
MPP8 and SETDB1 regulate common genes in mouse embryonic stem cells (mESCs). (**A**) Loss-of-function assays. HM1 ESCs were transfected with a scrambled control siRNA (Scr) or siRNA directed against *MPP8* or *SETDB1* mRNAs. The knockdown (KD) efficiency as assessed by WB is presented. Two independent experiments are shown as #1 and #2. (**B**) Volcano plot representations of deregulated genes as measured by RNA-seq in HM1 ESCs upon *MPP8* KD (Left panel) and upon *SETDB1* KD (Right panel). Red dots indicate significantly upregulated genes and blue dots indicate significantly downregulated genes (*p*-value < 0.05). (**C**) Upper panel: Venn diagram representation of upregulated genes upon *MPP8* or *SETDB1* KD (log2FC > 1 and *p*-value < 0.05). Lower panel: Gene ontology (GO) analysis of 190 overlapping upregulated genes. The top 5 biological processes are presented (*p*-value < 0.05). (**D**) Upper panel: Venn diagram representation of genes bound by MPP8 in mESCs and upregulated genes upon MPP8 KD (log2FC > 1 and *p*-value < 0.05). Lower panel: Gene ontology (GO) analysis of 42 overlapping genes. The top 6 biological processes are presented.

siRNA-mediated acute KD of *MPP8* or *SETDB1* in HM1 ESCs showed a 70% decrease in mRNA level (not shown) of the specific targets and about the same decrease at the protein level (Figure 3A).

RNA-seq analyses showed that 733 genes were dysregulated upon *MPP8* KD and 1605 upon *SETDB1* KD (Log2FC > 1 and *p*-value < 0.05) (Figure 3B and Supplementary Figure S3). Interestingly, a total of 190 genes were commonly upregulated upon *MPP8* or *SETDB1* KD (Figure 3C and Supplementary Table S4). Gene ontology analysis indicate that commonly upregulated genes are mainly involved in the regulation of cell differentiation, cell proliferation and, interestingly, telomere maintenance (Figure 3C and Supplementary Figure S3).

We observed that 112 genes that are bound by MPP8 were also upregulated upon *MPP8* KD (Figure 3D). Interestingly, important biological processes such as regulation of transcription or DNA replication seems to be affected upon *MPP8* KD (Figure 3D).

*SETDB1* or *MPP8* KD induced also gene expression downregulation (Figure 3B). While gene upregulation in the case of *SETDB1* and *MPP8* KD is expected, the downregulation is less. In the majority of cases downregulation is due to secondary events. Indeed, crossing the list of genes that are downregulated upon *MPP8* KD and bound by MPP8 showed only 20 genes which are bound by MPP8 and downregulated upon *MPP8* KD contrary to the 42 upregulated genes.

Altogether, our results suggest that MPP8 and SETDB1 not only are enriched in similar genome regions, but also co-regulate a set of genes.

### 3.4. SETDB1 and MPP8 Cooperates to Silence Satellite DNA Repeats

**Figure 4:**
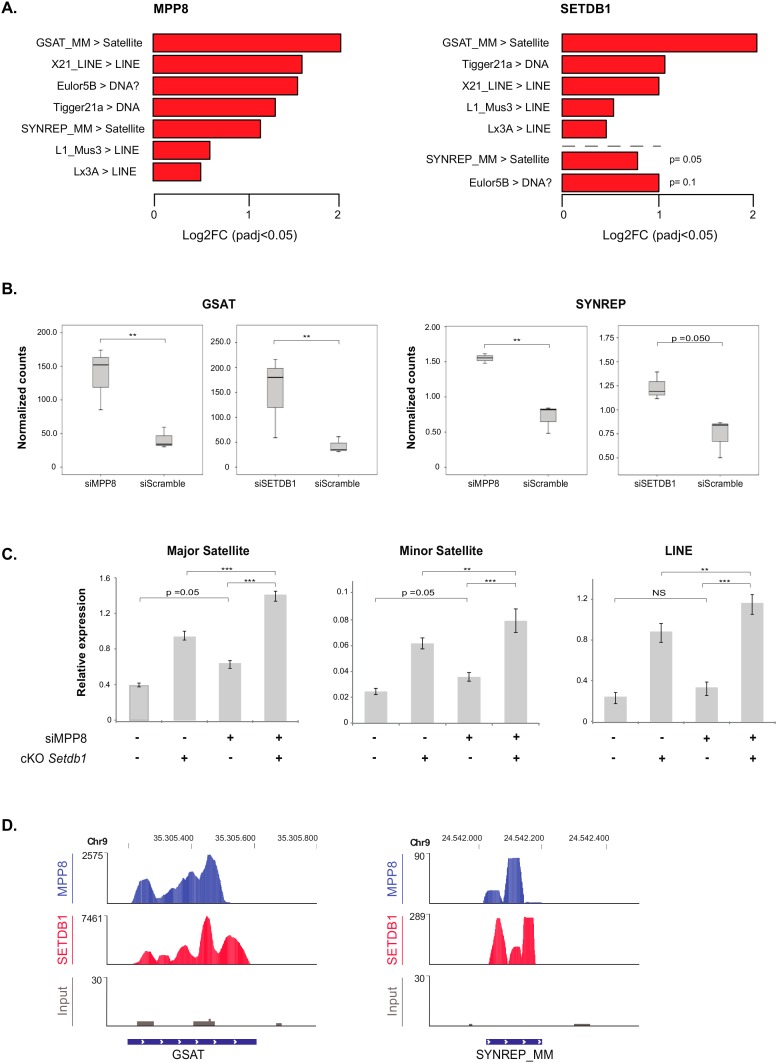
The major and minor satellites are de-repressed upon acute *MPP8* or *SETDB1* loss-of-function in mESCs. (**A**) Left panel: RNA-seq data showing the differential expression of repeated elements (Log2FC > 1; *p*-value < 0.05) in HM1 mESCS upon siRNA-mediated acute KD of *MPP8* and control. Right panel: Corresponding up-regulated repeated elements (*p*-value < 0.05) in HM1 mESCS upon siRNA-mediated acute KD of *SETDB1* and Scramble treatment. Note that SYNREP and Eulor5B sequences had *p*-value > 0.05. Red bars indicate upregulated repeated elements. (**B**) Barplot representation of normalized counts from RNA-seq data of the satellites GSAT and SYNREP in mESCs upon *MPP8* or *SETDB1* KD. n = 3 biological replicates ** *p* < 0.05. (**C**) RT-qPCR analysis of major and minor satellites sequences, as well as LINE repeats, in mESCs upon *MPP8* KD and/or *Setdb1* KO (cKO). mRNA levels were normalized to *GAPDH* or Cyclophilin A mRNA. For statistical significance, Student t-tests were applied to data following normal distribution, otherwise, Mann–Whitney’s unpaired tests were applied. (n = 3 biological replicates) ** *p* < 0.05 or *** *p* < 0.01. (**D**) Genome browser representation including tracks for MPP8 ChIP-seq and SETDB1 ChIP-seq in mESCs at GSAT and SYNREP satellites. Repeats data was retrieved from Repeat Masker via UCSC genome browser.

Concerning transposable elements, the differential expression compared to scrambled siRNA-transfected HM1 cells showed that the most de-repressed ones upon *MPP8* KD are GSAT and SYNREP satellites, L1, X21 and Lx3 LINE repeats (Figure 4A). Thus, our data showed for the first time that MPP8 is required in the silencing of satellite repeats in mESCs. Interestingly, the majority of these transposable elements were also upregulated upon *SETDB1* KD (Figure 4A and Supplementary Table S5). Of note, in accordance to previous reports [16,30], our results also show many families of ERV and LINE sequences upregulated after *SETDB1* KD (Supplementary Figure S4). Next, we checked the overlap with the *SETDB1* KD results. Interestingly, we found that the satellite repeats GSAT and SYNREP from the (peri)-centromeres are also de-repressed upon *SETDB1* KD (Figure 4B).

As shown above, MPP8 and SETDB1 independently regulate the expression of satellite DNA and LINE repeats. We thus tested whether the effect of MPP8 changes in presence or absence of SETDB1. To address this, we used conditional *Setdb1* KO TT2 mESCs (cKO) [16], in which endogenous SETDB1 depletion is totally achieved after 96 h of treatment with 4-hydroxy-Tamoxifen (OHT). We induced siRNA-mediated acute KD of *MPP8* and at the same time endogenous depletion of *SETDB1* in these ESCs, in parallel to the ad hoc controls. *MPP8* KD showed a 40% decrease in *MPP8* mRNA level when depletion of *SETDB1* was induced, and 67% decrease without OTH treatment. However, even though the efficiency of the *MPP8* KD was not very high, a trend toward significance showing upregulation of minor and major satellites and LINE repeats upon *MPP8* KD was observed, in accordance with our RNA-Seq data. Of note, the consensus sequence of the primers we used in RT-qPCR could amplify many families of satellite and LINE repeats, not only the specific ones that are upregulated in the RNA-seq. Interestingly, upregulation of major and minor satellites as well as LINE elements is significantly higher upon concomitant *MPP8* and *SETDB1* loss-of-function (Figure 4C), suggesting their synergistic roles.

Finally, the analysis of the MPP8 and SETDB1 ChIP-seq-associated reads confirmed a good recruitment on the GSAT and SYNREP repeats (Figure 4D).

Altogether, these data suggest that SETDB1 and MPP8 cooperate in satellite DNA repeats silencing in mESCs.

## 4. Discussion

We have combined biochemical and genomic strategies to get insights on the functions of the major H3K9 methylation reader MPP8 in mESCs. We first characterized the MPP8 complex partners and confirmed that MPP8 form complexes with many H3K9 methylation machinery members, including G9A and GLP H3K9 KMTs, as already known. Interestingly, we found that MPP8 interacts more robustly with the H3K9 KMT SETDB1 and its co-factor ATF7IP. We showed a physical and functional interaction between MPP8 and the major H3K9 KMT SETDB1 in mESCs. *SETDB1* loss-of-function induces early embryonic lethality between 3.5 and 5.5 dpc [7]. Indeed, H3K9 methylation established by SETDB1 is essential in pluripotent mESCs where, in addition to coding gene repression, it is required for repetitive elements silencing ensuring the stability of genetic information. In general, H3K9 methylation and its effectors play key roles in the non-coding genome and transposable elements silencing in pluripotent ESCs compared to more committed cells in which an additional layer of DNA methylation is established [10,31,32].

Our data showed that in addition to co-regulating coding genes, the association of the H3K9 KMT SETDB1 with the H3K9 methylation reader MPP8 extend to more genomic elements, including major and minor satellite DNA as well as some LINE elements.

The *MPP8* gene is present only in vertebrates, reminiscent of some other proteins that are linked to the H3K9 methylation epigenetic mark such as TRIM28/KAP1 and ATF7IP (the SETDB1 cofactor), while some others are not that conserved, such as Heterochromatin Proteins HP1. MPP8 has been proposed to be involved in “position-effect variegation” (PEV)-like in the human and mouse genomes in the HUSH complex [33]. MPP8 has been described to repress the expression of a retrovirus incorporated into heterochromatin with a screen using siRNAs [33]. In vertebrates, none of the HP1 proteins seemed to induce a PEV-like phenomena on integrated reporter in human cells [34], two proteins of the HUSH complex do, SETDB1 and MPP8. This evolution in the H3K9 methylation by SETDB1 and its readers from fly to vertebrates is concomitant to the apparition of new factors MPP8, MCAF, KAP1 and a whole new zinc finger family of proteins.

Expression of *MPP8* is especially high in stem and germ cells and thus may play key roles in these cells chromatin features. As H3K9 methylation is mainly located in repeated elements, so do the main H3K9 KMTs and effector proteins that bind these motifs. Among these repetitive elements, the major and minor satellite DNA, the LTR-containing retroelements ERVs and the non-LTR-containing retrotransposons LINE, are epigenetically repressed by H3K9 methylation in ESCs [2,35], which then constitute a docking site for effector proteins which bind methylated H3K9 via specific domains, such as MPP8 via its chromodomain.

Lysine methylation is not known to directly regulate chromatin structure, since addition of a methyl group to a lysine does not affect the charge, in contrast to acetylation for example [36]. Instead, methylated lysines are recognized by effector proteins called readers of lysine methylation, such MPP8 that binds methylated H3K9 via its chromodomain, and regulate chromatin structure and the subsequent biological response. Thus, the combined recruitment of a writer and a reader of lysine methylation, for instance SETDB1 and MPP8, would provide a mean for not only the establishment but also the spreading and maintenance of H3K9me3 at the targeted genomic regions, such as at repeated elements. MPP8, through its interaction with SETDB1, would participate in the spreading of the SETDB1-mediated H3K9 trimethylation to silence the transcription of repeated elements. In addition, MPP8 has been shown to bind the G9A-methylated form of the SETDB1 cofactor ATFIP7 [37], thus providing another mean to further stabilize the writer-reader interaction.

In the mouse, the centromeric and pericentromeric regions are enriched by two conserved tandem repeats, which are the minor and major satellite DNA (such as the SYNEREP_MM and GSAT_MM, respectively) [38,39]. These satellite sequences are important for sister chromatid cohesion, kinetochore formation and spindle microtubule attachment during M-phase. Major and minor satellite repeats are transcribed during the cell cycle and during early development [39,40,41,42]. Interestingly, MPP8 is known to be phosphorylated at the M-phase of the cell cycle and its phosphorylation lowers its affinity towards H3K9me3 [14,43,44]. This is concomitant to the H3S10 massive phosphorylation during M-phase [45,46]. Thus, these two mechanisms could contribute to the satellite DNA transcription during the cell cycle, which is important for heterochromatin re-establishment during cell division.

In summary, our data suggest that SETDB1 and MPP8 cooperate in the cell cycle-dependent repression of the satellite DNA.

## Supplementary Materials

The following are available online at https://www.mdpi.com/2073-4425/10/10/750/s1, Figure S1: (A) Diagram of the experimental plan for MPP8 complex characterization. (B) Methylation states of H3K9, and H3K36 associated to MPP8 versus the H3K9 KMT G9A. nm, non-modified; me, monomethyl; me2, dimethyl; me3, trimethyl. Results showed are the mean of two independent experiments. Figure S2: (A) Upper panel: Venn diagram representation of genes bound by MPP8 and/or SETDB1 in mESCs Lower panel: Gene ontology (GO) analysis of 1329 overlapping genes. The top 10 biological processes are presented. (B) Genome browser representation including tracks for MPP8 ChIP-seq and SETDB1 ChIP-seq in mESCs at *Cldn34d* coding gene. Figure S3: (A) Gene ontology (GO) analysis of 396 upregulated genes in mESCs upon *MPP8* KD (Log2FC > 1; *p*-value < 0.05). (B) Gene ontology (GO) analysis of 1050 upregulated genes in mESCs upon *SETDB1* KD (Log2FC > 1.5; *p*-value < 0.05). Figure S4: Fold changes of individual transposons upregulated upon *SETDB1* KD (Log2FC > 1; *p*-value < 0.05) are shown in a barplot. Table S1: MPP8 complex components as revealed by mass spectrometry in HeLa cells. Table S2: Genes enriched in the binding sites of MPP8 and SETDB1 in mESCs. Table S3. Transposable elements enriched in the binding sites of MPP8 and SETDB1 in mESCs. Table S4: Upregulated genes identified by comparing RNA-seq data from biological triplicates upon MPP8 and SETDB1 KD in mESCs (Log2FC > 1; *p*-value < 0.05). Table S5: Upregulated transposable elements identified by comparing RNA-seq data from biological triplicates upon MPP8 and SETDB1 KD in mESCs (Log2FC > 0.5; *p*-value < 0.05).
